# Towards Better Understanding of the Pathogenesis of Neuronal Respiratory Network in Sudden Perinatal Death

**DOI:** 10.3389/fneur.2017.00320

**Published:** 2017-07-06

**Authors:** Riffat Mehboob, Mahvish Kabir, Naseer Ahmed, Fridoon Jawad Ahmad

**Affiliations:** ^1^Biomedical Sciences, King Edward Medical University, Lahore, Pakistan; ^2^Faculty of Allied Health Sciences, University of Lahore, Lahore, Pakistan; ^3^Department of Chemistry, School of Science, University of Management and Technology (UMT), Lahore, Pakistan; ^4^Department of Cardiac Surgery, University of Verona Medical School, Verona, Italy; ^5^Section of Pharmacology, University of Verona Medical School, Verona, Italy

**Keywords:** sudden infant death, sudden fetal death, sudden perinatal death, sudden intrauterine death, stillbirth, neuropathology

## Abstract

Sudden perinatal death that includes the victims of sudden infant death syndrome, sudden intrauterine death syndrome, and stillbirth are heartbreaking events in the life of parents. Most of the studies about sudden perinatal death were reported from Italy, highlighting two main etiological factors: prone sleeping position and smoking. Other probable contributory factors are prematurity, male gender, lack of breastfeeding, respiratory tract infections, use of pacifiers, infant botulism, extensive use of pesticides and insecticides, etc. However, extensive studies across the world are required to establish the role of these factors in a different subset of populations. Previous studies confirmed the widely accepted hypothesis that neuropathology of the brainstem is one of the main cause of sudden perinatal death. This study is an effort to summarize the neuropathological evaluation of the brainstems and their association to sudden perinatal death. Brainstem nuclei in vulnerable infants undergo certain changes that may alter the sleep arousal cycle, cardiorespiratory control, and ultimately culminate in death. This review focuses on the roles of different brainstem nuclei, their pathologies, and the established facts in this regard in terms of it’s link to such deaths. This study will also help to understand the role of brainstem nuclei in controlling the cardiorespiratory cycles in sudden perinatal death and may provide a better understanding to resolve the mystery of these deaths in future. It is also found that a global initiative to deal with perinatal death is required to facilitate the diagnosis and prevention in developed and as well as developing countries.

## Introduction

Sudden perinatal mortalities include sudden fetal death or Sudden Intrauterine Death Syndrome (SIUDS), stillbirths, and Sudden Infant Death Syndrome (SIDS) due to some unknown reason. Stillbirth is death of a fetus after 20 weeks of gestation, weighing 350–1,000 g (1). The annual global incidence of stillbirths is 2.7 billion, with 15–35% more deaths in developing countries, which is very alarming (2, 3). SIDS also termed as “Crib death” or “Cot death” is defined as the sudden and inexplicable death of an apparently healthy newborn or infant who dies before the first birthday and reason remains a mystery even after a complete autopsy or thorough investigation (4). To find out the exact cause of death in SIDS or SIUDS, victims are a major diagnostic challenge. SIUDS are broadly categorized as accidental and non-accidental mortalities (5). It was found that victims of sudden perinatal deaths usually belong to economically poor family and incidence is high in winter, during midnight and weekends (6–8). Many other risk factors were also observed in the SIDS victims such as male gender (9), ethnicity (10), and deformational plagiocephaly (11). Some maternal factors reported were maternal age (12), obesity (13), and smoking during pregnancy (14), whereas environmental factors were prone sleeping position (15), soft bedding, over heating (16), lack of breastfeeding (17), and higher latitudes (18). More recently, some new theories have been proposed, and it was highlighted that infant gut microbiome may modulate the brainstem serotonergic system and may serve as a new possible risk factor for causing SIDS (19). Latest theories like SIDS-critical diaphragm failure hypothesis suggest that the critical diaphragm failure during pregnancy may end up in SIDS by cessation of breathing (15), whereas substance P–neurokinin 1 hypothesis suggests a possible involvement of this tachykinin peptide in sudden perinatal deaths by modulating the cardiorespiratory control (20).

The causes of these unexplained deaths can be environmental, genetic or congenital, etc. So far, the most accepted hypothesis to define SIDS is triple-risk model of Filiano and Kinney (21), in which the infants exposed to external stress, and have some intrinsic vulnerability will be at higher risk of having neurological and developmental abnormalities that can result in SIDS (22). The National Institute of Child Health and Development SIDS Strategic Plan 2000 states that “SIDS is a developmental disorder. It’s origins are during fetal development” (23). Subtle hippocampus abnormalities, seizures, malfunctioning in central nervous system mechanisms, abnormalities in neurotransmitter secretions, and in the nuclei of brainstem cells are also suggested as causes of SIDS (24).

## Neuropathology of Sudden Perinatal Death

Neuropathology deals with the diseases of the nervous system tissue, either through small surgical biopsies or whole-body autopsies. Neuropathological studies include anatomy, pathology, neurology, and neurosurgery (4). In this study, the main focus is on summarizing the neuropathological anomalies of sudden fetal deaths, stillbirths, and infant mortalities due to alterations in neurotransmitter’s release and nuclei of brainstem neuronal centers. The human cerebellar cortex development involves rapid transformations, thickness, as well as the reorganization of cortical layers in the fetal and early postnatal stages (25). Any change due to mutations, epigenetic and environmental factors such as smoking, hypoxia, pesticide exposure, and infection can result in neuropathological conditions. Even though current studies are unable to pin point the causes but brainstem abnormalities that are responsible for respiration and responses to asphyxia, especially in the sleep and arousal, are thought to be the probable causes (26, 27). Defects in brainstem neural circuits involved in cardiorespiratory regulation may be one of the leading causes of SIDS (28).

## Brainstem Control of Respiration During the Transition from Water to Air Breathing

Breathing rhythm in fetus begins at the 10th week of gestation (29) which changes from irregular to a regular pattern at the time of birth by unknown mechanisms. In the neonatal period, a regular respiratory rhythm (RR) and cardiorespiratory coupling is controlled by neuronal centers in the brainstem (30). These RRs are controlled by several pathways in the neuronal networks, e.g., pre-Botzinger complex and the Kölliker–Fuse as well as some cortical and cerebellar networks (31). These pathways are also involved in involuntary functions, sleep–awake cycle, and upper respiratory tract reflexes. It is found that brain-derived neurotrophic factor (BDNF) is involved in steady rhythm generation.

In response to stress such as hypoxia, these networks are able to reconfigure, to generate multiple breathing patterns, and to facilitate autoresuscitation. There are vital changes in caudal serotonergic (5-HT) system at the end of the fetal period and the start of the neonatal period that are regulated by neuronal networks. Serotonin (5-HT) receptor binding is gradually decreased as the gestation progresses.

Instability in the early control of breathing is proportional to frequency of apnea in infants. Brief apneic spells are common within the first few minutes after birth, later on more prolonged episodes of apnea are observed. These apneic episodes (breath holding) are associated with prematurity, laryngeal chemoreflex activity or bradycardia, and loss of muscle tone (“near-miss SIDS” or apparent life-threatening events) (32). Episodic apnea and bradycardia have been observed in the infants who died of SIDS (33). Vulnerable infants with immature neuronal centers are unable to face the life-threatening challenges such as hypoxia and hypercapnia during sleep, which may lead to imbalances in serotonergic networks (34). Consequently, abnormality in specific brainstem neuronal networks have been observed in SIDS that cause failure of these reflex responses to arousal.

### Pontine Kölliker–Fuse Nucleus (KFN)

**Figure 1:**
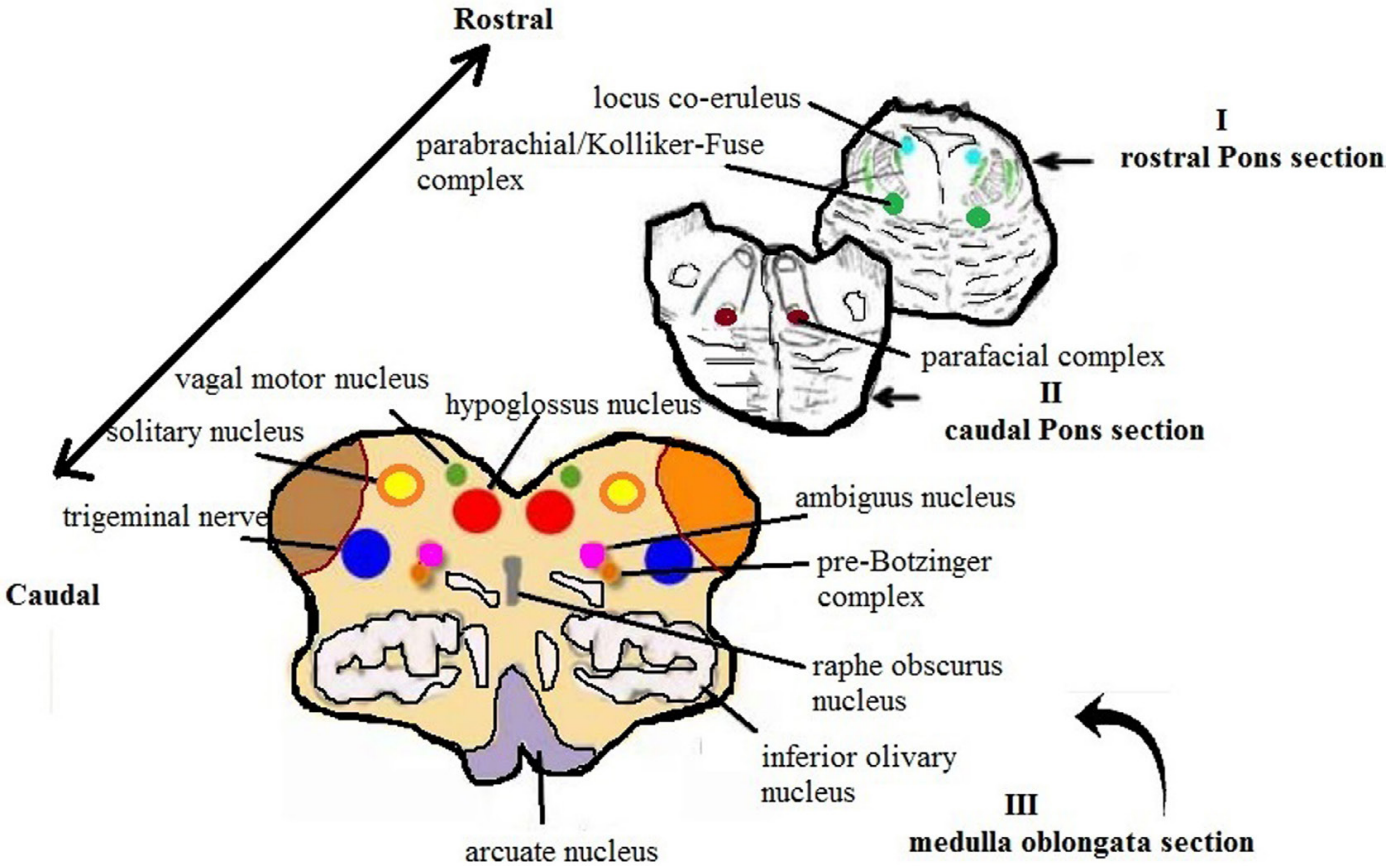
Schematic representation of the main histological sections obtained from the brainstem for the anatomopathological examination [modified image adapted from Matturri et al. (31)].

Studies established the role of pontine KFN (Figure 1; Table 1) in breathing control; it is interconnected with the prevalent serotonin and noradrenaline neurons in the brainstem. It is suggested that orexin has a strong effect on the brainstem raphe nuclei (RN) and locus coeruleus, in arousal from sleep (35). The neurobiological functions of these stem cell nuclei are closely linked to the breathing modifications (36). As KFN is a main component of the orexin system that is involved in arousal, KFN was observed to play a key role in providing a breathing rhythm and coordination of sleep-to-wake transition. Any defect in orexin expression in KFN is responsible for prevention of arousal and can be a crucial factor in causing SIDS (36).

**Table 1:** Summary of studies on brainstem nuclei along with their physiological and pathological roles (+ indicate increase and − is decrease in expression).

Nucleus	Brain area	Role of nucleus	Neurotransmitter	Expression	Alteration in function	Possible cause	Reference
KFN	Rostral PONS, brainstem	Arousal/sleep breathing control in perinatal life, synaptic plasticity	OR, BDNF	−	Fetal inhibitory reflex arrest breathing, deranged normal KFN development, and loss of breathing control	Hypoxic conditions, smoking	(36, 37)
ICN	Mesencephalon	Acoustic processing	5-HT	−	Dysgenesis of RN, superior ON, ICN	Nicotinic absorption, smoking	(26, 38)
Nucleolus	Brainstem	Ribosomal synthesis	AgNOR	−	PC degeneration, disturbed cardiac cycle	Nicotinic absorption, smoking	(39)
AP	Fourth ventricle	Controls vomiting	−	−	AP lesion	Insecticide	(40)
LC, KFN, CAN, RN, pre-BotC, PF/FC	Cerebral cortex	Breathing control, sleep–awake cycle	α7-NAcR	+	Hypoplasia of all nuclei	Smoking, insecticide	(14, 41)
POD	Cerebellar Purkinje	RR	α7-NAcR		Alterations of POD network	Smoking	(42)
NN	Brainstem	Mitotic cycle	NeuN	−	Cell death increased, neuronal immaturity	Smoking	(43)
LC	Brainstem	Sleep–wake cycle, control of CRS	TK, NM, TH	−	NM, hypoplasia, neuronal death, alterations of noradrenaline system, low neuromelanin, neuronal death	Smoking	(44)
SOC	Brainstem	Acoustic information	−	−	Hypoplasia of ON, RTN, FN, hypercellularity, dysgenesis of structures related to RR, alterations in auditory, and respiratory network	Smoking	(26, 45)
RTN	Caudal pons	Breathing, chemoreception	PHOX2B	−	Developmental abnormalities in RTN	Smoking	(46)
AP	Brainstem, fourth ventricle choroid plexus	Autonomic control of cardiac and respiratory activity	−	−	Lack of vascularization, hypoplasia, cystic formations, reactive gliosis	Smoking	(47)
STrN	Brainstem	Pain, thermofluctuations, RR	SP	−+	Pre-BtzC, RN, and AN hypoplasia	Smoking	(48)
IMN	Brainstem	Breathing activity	−	−	Hypoplasia, neuronal immaturity	Smoking	(49)
G-Mt	Brainstem	Modulation of spinal cord motor activity	−	−	Hypoplasia, apoptosis	Smoking	(50)
HGN	Brainstem	Swallowing, chewing, vocalization, inspiration	SM	+	Hypoplasia, hyperplasia, no interneurons	Smoking	(51)
RN	Brainstem	Sleep–wake cycle	5-HTT	−	Hypoplasia	Smoking	(52)
Pre-BotC	Medulla	RR	NK1R, SM	−	Hypoplasia, low neuronal no., dendritic hypodevelopment	Smoking	(53)

*OR, orexin receptor; BDNF, brain-derived neurotrophic factor; 5-HT, serotonin; AP, area prostrema; RTN, retrotrapezoid nucleus; ON, olivary nucleus; LC, locus coerulius; STrN, spinal trigeminal nucleus; KFN, Kölliker–Fuse nucleus; ICN, inferior collicus nucleus; RN, raphe nucleus; AN, arcuate nucleus; PF/FC, parafacial/facial complex; pre-BotC, pre-Bötzinger; IMN, intermediolateral nucleus; G-Mt, Guillain–Mollaret triangle (dentato-rubro-olivary network); HGN, hypoglossal nucleus; α7-NacR, α7-nicotinic acetylcholine receptor; TK, tyrosine kinase; NM, neuromelanin; CRS, cardiorespiratory system; RR, respiratory rhythm; TH, tyrosine hydroxylase; NN, nucleus of neurons; POD, Purkinje-olivo-dentate network; SM, somatostatin; 5-HTT, serotonin transporter; NK1R, neurokinin 1 receptor*.

Experimental studies indicate that the neurotrophin BDNF has a vital role in the central respiratory network development to sustain life. In the prenatal and postnatal breathing circuit, pontine KFN is a fundamental component (54). BDNF pathway dysfunctions may possibly distort the normal KFN development in SIUDS and SIDS victims by interfering with the breathing control. Alterations in the BDNF expression in KFN have been observed in many respiratory diseases in human such as the Rett’s and the congenital central hypoventilation syndromes (37).

### Inferior Colliculus Nucleus

Developmental defects of hearing pathways involve defects in the specific brainstem centers, specifically in the cochlear, vestibular, superior olivary, and inferior olivary complex (Figure 1). Significantly, more alterations were observed in cytoarchitecture of auditory and respiratory networks of SIDS cases as compared to controls in one study (26). The inferior colliculus has a vital role in the processing of acoustic information. It is believed that neuromodulator serotonin concentration can be a factor in sudden unexplained fetal and infant death syndromes. Weak serotonin positivity was observed in a study conducted on brainstems of SIDS and SIUDS victims, indicative of functional abnormality of inferior colliculus. Hypoplasia or anomalies in the associated structures, e.g., RN and the superior olivary complex was also observed in the fetus of smoking mothers (Table 1). A role of inferior colliculus in breathing apart from hearing was also suggested (38).

### Locus Coeruleus Complex

Locus coeruleus complex (Figure 1; Table 1) is a part of the brainstem in pons mainly responsible for the physiological responses to conditions of stress and panic. It is the main region that produces norepinephrine (noradrenaline), tyrosine hydroxylase, and neuromelanin (NM) (55). A strong correlation between defects in noradrenaline system, low levels of NM, hypoplasia, along with a high neuronal death rate, were found mainly in the locus coeruleus complex of fetal and infant sudden death victims (44). Studies have shown that locus coeruleus complex is involved in vital activities related to the brain interconnections and behavioral adjustments, including coordination of the sleep–wake cycle and control of the cardiorespiratory functions (56).

### Superior Olivary Complex

The superior olivary complex (Figure 1; Table 1) is a group of brainstem nuclei that have multiple roles in hearing and is involved in ascending and descending auditory pathways (57). Irregular cytoarchitectural patterns like hypoplasia/agenesis, immature hypercellularity, and dysgenesis of contiguous structures involved in breathing circuit in medial superior olivary nucleus were reported in a study, and it was proposed that this nucleus had influence on all the vital activities along with hearing (45).

### Retrotrapezoid Nucleus (RTN)

The RTN is part of caudal pons and comprises cluster of glutamatergic and non-aminergic neurons that are responsible for the homeodomain transcription factor Phox2b (a transcriptional factor involved in congenital central hypoventilation syndrome) expression (58). Immunohistochemical expression of Phox2b neurons inside the caudal pons points out the developmental abnormalities of the human RTN (Table 1). It may acutely affect the chemoreception control, thus, performing a vital part in the pathogenesis of SIUDS and SIDS (46).

### Spinal Trigeminal Nucleus (STrN)

**Figure 2:**
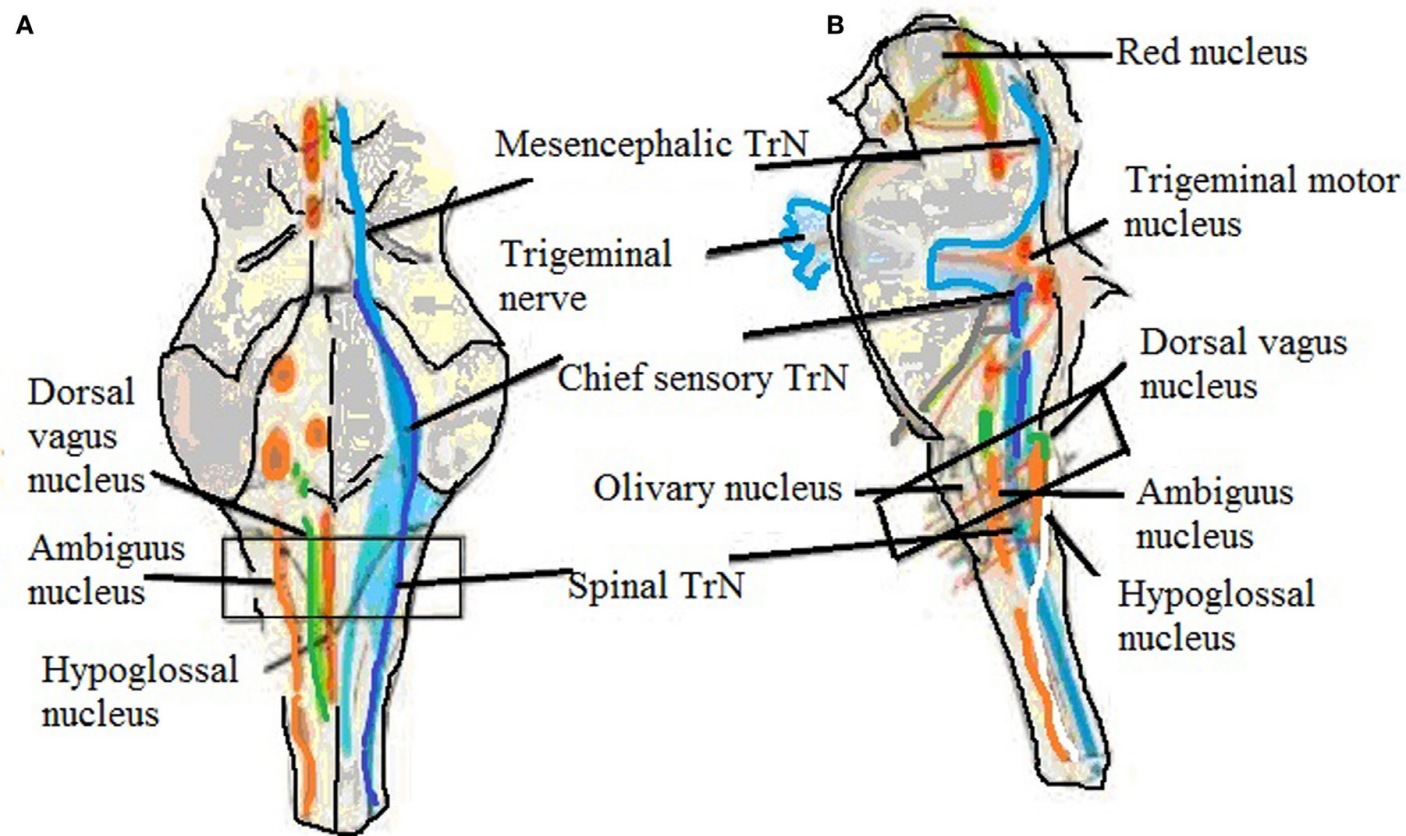
Brainstem showing trigeminal nucleus and also shows the level of sampling. **(A)** Ventral view and **(B)** side view showing trigeminal nerve, mesencephalic, chief sensory, and spinal trigeminal nucleus (60).

The STrN (Figure 2; Table 1) is part of medulla, and it transmits information related to pain and temperature in the orofacial region. The cranial nerves transmit pain stimuli from peripheral regions to the STrN (59). A reduced SP expression levels in the fibers of STrN in SIDS victims and higher levels in SIUDS victims were observed (20, 48).

### Intermediolateral Nucleus (IMN)

In the brain, the sympathetic preganglionic neurons reside in the IMN that is a part of spinal cord. These are groups of columnar cells organized longitudinally, in the gray matter of the lateral horn. These cells are present between the first thoracic spinal region and the third lumbar region (61). Experimental studies have demonstrated the role of IMN in the breathing activities and development of a spinal cord–brainstem network (62). In SIDS, IMN fails to mature progressively; it’s neurons do not transform from a round to a polygonal shape with extended axons and drastically decrease in number. In unexplained fetal and infant death victims, hypodevelopment of IMN such as neuronal immaturity in a normal structure, hypoplasia, and agenesis was seen (49) (Table 1).

### Guillain–Mollaret Triangle (G-Mt) (Dentato-Rubro-Olivary Network)

**Figure 3:**
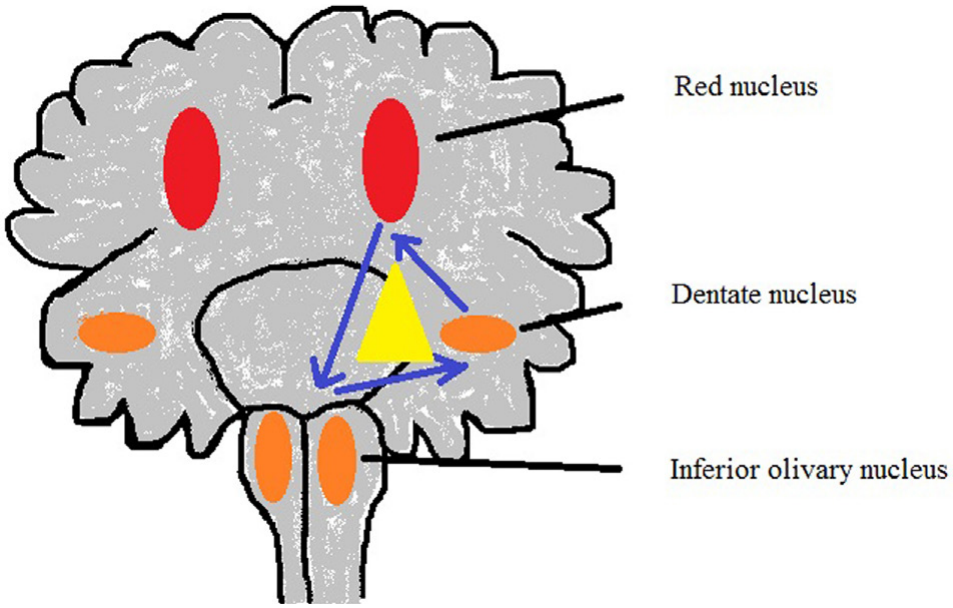
Sketch of Guillain–Mollaret triangle derived from Lavezzi et al. (50).

The G-Mt (Figure 3; Table 1) has three parts: the ipsilateral red nucleus, the inferior olive, and the contralateral dentate nucleus in the midbrain, medulla, and cerebellum to form dentato-rubro-olivary pathway (63). G-Mt is known to be involved in the pathogenetic mechanisms of the palatal myoclonus, in SIDS and SIUDS. A significant increase of lesions of these three nuclei were found in SIDS victims (50).

### Medullary Hypoglossal Nucleus (HGN)

The hypoglossal nerve is a motor nerve that controls extrinsic and intrinsic muscles of the tongue. It arises from the HGN (Figure 1; Table 1) in the brain stem and controls swallowing, chewing, vocalization, and inspiration (64). HGN anomalies such as hypo/hyperplasia, somatostatin positivity, and absence of interneurons were evident in SIDS cases (51). Unlike to the trigeminal nucleus, HGN is not considered as a main respiratory regulatory center, yet, it contains motoneurons with respiratory-related rhythmical discharges. Primarily, HGN controls the genioglossus, an extrinsic muscle of the tongue, which plays a significant role in regulating a patent airway during inspiration (65, 66).

### Raphe Nuclei

The RN (Figure 1; Table 1) are medial part of the reticular formation that forms a crest of cells in the center and in the medial portion of the brainstem (67). In a study, cytoarchitecture and the localization of human RN in the brainstem were done to analyze the association of raphe nucleus pathology and serotonin transporter gene (5-HTT) polymorphisms. It was also suggested that SIUDS should not be viewed separately from SIDS, due to potentially shared neuropathological and genetic grounds (52).

### Pre-Bötzinger Complex (Pre-BötC)

In the ventrolateral medulla of the brainstem, a cluster of interneurons is present known as pre-BötC (Figure 1; Table 1). It is believed that it has a vital role in the generation of RR in humans (68). Neuropathology of the pre-BötC, altered neurokinin 1 receptors, and somatostatin expression were observed in a subset of SIDS and SIUDS victims as compared to the controls. Hypoplasia with a low neuronal number with dendritic hypodevelopment, defective neuronal morphology, immunonegativity of neurotransmitters, and agenesis was sighted. These abnormalities are directly linked with the neonatal deaths and still births (53).

In most of these studies, an association has been found with maternal smoking. Nicotine is one of the few lipid-soluble substances that are able to go beyond the blood–brain barrier (69) and act directly on the expression of genes that control the developing brain. Therefore, among the numerous compounds present in cigarette smoke, carbon monoxide and nicotine could affect the fetal brain through indirect or direct action (70). As there are not many studies conducted on SIDS and SIUDS worldwide and it is multifactorial, so we cannot conclude concretely, that only smoking is the main etiological factor. Recently, some studies have been done on the role of pesticides and insecticides in these sudden deaths and an association has been observed (41, 71). Most of these studies were conducted mainly in Italy, so there is a need to explore the risk factors in other parts of the world too, e.g., Southeast Asia where infant mortality rate is very high and population is exposed to extra risk factors like consumption of banned insecticides like DDT among others. Moreover, there is no epidemiological data available regarding SIDS and SIUDS in these regions.

## Conclusion

Neuropathology in brainstems of SIDS and SIUDS victims are summarized in this study. It is found that several alterations in the brain centers possibly lead to sudden deaths. This updated effort will help in better diagnosis and identification of such cases. Moreover, an association with maternal smoking has been observed in the reported studies. It is noticed that sufficient data to establish all causative factors is not available. So, there is a need to study other dimensions to find out the etiological factors in different populations and different regions of the world. There is an urgent need to expand these studies in other regions of the world, particularly in South East Asia where health-care facilities are very poor and banned agricultural pesticides are still in use.

## Author Contributions

RM conceived the idea, planned the review manuscript, made some figures, helped in writing the manuscript, and finalized it. MK gathered already existing literature in the field, made some figures, and helped in writing some parts of manuscript. NA made the table and helped in writing the manuscript. FA finalized the manuscript. All the authors have revised, checked, and approved the final version of the manuscript.

## Conflict of Interest Statement

The authors declare that the research was conducted in the absence of any commercial or financial relationships that could be construed as a potential conflict of interest.
