# Supplementary material for: Towards Better Understanding of the Pathogenesis of Neuronal Respiratory Network in Sudden Perinatal Death
Source: Front Neurol. 2017 Jul 6;8:320. doi: 10.3389/fneur.2017.00320 (PMC5498558; doi:10.3389/fneur.2017.00320)
Supplement: Supplementary file 1 [file table_1.doc]

| **Nucleus** | **Brain area** | **Role of nucleus** | **Neurotransmitter** | **expression** | **Alteration in function** | **Possible cause** | **Reference** |
| --- | --- | --- | --- | --- | --- | --- | --- |
| KFN | Rostral PONS, brainstem | Arousal/sleep  breathing control in perinatal life, Synaptic Plasticity | OR, BDNF | _ | fetal inhibitory reflex arrest breathing, deranged normal KFN development and loss of breathing control | hypoxic conditions, smoking |  |
| ICN | mesencephalon | Acousting processing | 5-HT | _ | dysgenesis of RN, superior ON, ICN | Nicotinic absorption ,smoking |  |
| Nucleolus | brainstem | Ribosomal synthesis | AgNOR | _ | PC degeneration, disturbed cardiac cycle | Nicotinic absorption, smoking |  |
| AP | 4th ventricle | controls vomiting | - | - | AP Lesion | insecticide |  |
| LC, KFN, CAN, RN, pre-BotC, PF/ FC | Cerebral cortex | Breathing control, sleep awake cycle | α7-NAcR | + | Hypoplasia of all nuclei | Smoking, insecticide |  |
| POD | Cerebellar purkinje | Respiratory rhythm | α7-NAcR | . | alterations of purkinje-olivo-dentate network | smoking |  |
| NN | brainstem | Mitotic cycle | NeuN | _ | Cell death increased, neuronal immaturity | smoking |  |
| LC | brainstem | Sleep-waking cycle, control of CRS | TK, NM, TH | _ | NM, hypoplasia, neuronal death, alterations of noradrenaline system, low neuromelanin, neuronal death | smoking |  |
| SOC | brainstem | Acoustic information | - | - | Hypoplasia of ON, RTN, FN, hypercellularity, dysgenesis of structures related to RR, alterations in auditoary and respiratory network | smoking |  |
| RTN | Caudal pons | Breathing, chemoreception | PHOX2B | - | Developmental abnormalities in RTN | smoking |  |
| AP | Brainstem, 4^th^ ventricle choroid plexus | Autonomic control of cardiac and respiratory activity | - | - | Lack of vascularization, hypoplasia, cystic formations, reactive gliosis | smoking |  |
| STN | brainstem | Pain. Thermofluctuations, respiratory rhythm | SP | _+ | Pre-BtzC , RN and AN hypoplasia | smoking |  |
| IMN | brainstem | Breathing activity | - | - | Hypoplasia, neuronal immatuarity | smoking |  |
| G-Mt | brainstem | Modulation of spinal cord motor activity | - | - | Hypoplasia, apoptosis | smoking |  |
| HGN | brainstem | Swalloing, chewing, vocalization, inspiration | SM | + | Hypoplasia, hyperplasia, no interneurons | smoking |  |
| RN | brainstem | Sleep wake cycle | 5-HTT | _ | hypoplasia | smoking |  |
| Pre.BotC | medulla | Respiratory rhythm | NK1R, SM | _ | Hypoplasia, low neuronal no., dendritic hypodevelopment | smoking |  |

Table 1: Summary of studies on brainstem nuclei along with their physiological and pathological roles (+ indicate incease and - is decrease in expression

**Abbreviations:**

Orexin receptor, OR; brain derived neurotrophic factor, BDNF; serotonin, 5-HT; Area prostrema; AP, Retrotrapezoid nucleus; RTN, Olivary nucleus; ON, Locus coerulius; LN, Spinal trigeminal nucleus; STrN, Kolliker-fuse; KFN, Inferior collicus nucleus; ICN, Raphe nucleus; RN, Arcuate nucleus; AN, Parafacial/ Facial complex; PF/FC, pre-Bötzinger;Pre- BotC, intermedio lateral nucleus IMN, Guillain-Mollaret Triangle (Dentato-Rubro-Olivary Network) (G-Mt),hypoglossal nucleus (HGN); α7-nicotinic acetylcholine receptor;α7-NacR, Tyrosine kinase (TK), neuromelanin (NM), Cardiorespiratory system (CRS), respiratory rhythm (RR), Tyrosine hydroxylase (TH); Nucleus of neurons, NN; purkinje-olivo-dentate network, POD; somatostatin, SM; Serotonin transporter, 5-HTT,;neurokinin 1 receptor, NK1R
